# Advancing Adolescent HIV Prevention and Care Through Implementation Science: An Agenda for Combating the Global AIDS Epidemic in Sub-Saharan Africa

**DOI:** 10.1007/s10461-023-04049-5

**Published:** 2023-03-24

**Authors:** Eric Goosby, Judith N. Wasserheit, Roger Glass, Peter Kilmarx

**Affiliations:** 1 Institute for Global Health Sciences, University of California, San Francisco, USA; 2 School of Medicine, University of California, San Francisco, USA; 3 School of Public Health, University of Washington, Seattle, USA; 4 Fogarty International Center, National Institutes of Health, Bethesda, USA

**Keywords:** Implementation science, HIV, Africa, Adolescent, Young adult

## Abstract

AIDS continues to be a major driver of adolescent mortality in Sub-Saharan Africa. Despite evidence of efficacy in this population, many efforts to address adolescent HIV have had limited impact across the region because of difficulty with implementation. The field of implementation science holds promise for addressing these challenges. The Fogarty-led Adolescent HIV Prevention and Treatment Implementation Science Alliance (AHISA) creates a platform for bidirectional learning between researchers and the users of research evidence that promotes the use of implementation science to strengthen adolescent HIV prevention and care across Africa. The unique contributions of AHISA are reflected in this supplement’s articles which represent the collective learning of the Alliance; illustrate the value of implementation science in the context of adolescent HIV; and identify critical research gaps that should be addressed by implementation science in the future.

More than two million adolescents are living with HIV globally. Although the overall number of HIV-related deaths worldwide is down 30% since the peak in 2006, estimates suggest that HIV deaths among adolescents are rising [1]. Moreover, data indicate that less than 25% of adolescents and young adults (AYA) know their HIV status [1]. Despite evidence of efficacy in this population, many efforts to address adolescent HIV have had limited impact in low-resource settings like sub-Saharan Africa (SSA) [2–4]. This is due, in part, to the unique developmental stages and age-related needs of AYA and the resulting multifaceted interpersonal, cultural, structural, and systems-level barriers that they encounter [5, 6]. The impact of effective interventions has also been limited because of lack of sustained funding and inconsistent or unsuccessful implementation, scale-up, and sustainability.

Implementation science offers a rigorous methodological approach to address these challenges. The Fogarty Adolescent HIV Prevention and Treatment Implementation Science Alliance (AHISA) leverages this rapidly developing field through a creative and impactful platform that promotes the use of implementation science to strengthen AYA HIV prevention and care in SSA. Formally launched in 2017 [7], AHISA consists of teams of researchers funded by the US National Institutes of Health, program implementers, AYA, and policymakers working in 11 countries in SSA. The Alliance was established to facilitate improved use of scientific evidence in AYA HIV programming; accelerate development of sustainable collaborations among researchers, implementers, and policy makers; and ensure that research is country driven and responsive to the local and adolescent context. AHISA supports in-person and virtual meetings and trainings as well as small collaborative contracts that aim to catalyze long-term region- and country-specific collaborations that address local implementation issues related to AYA and build implementation science capacity across SSA [8].

The unique contributions of AHISA are reflected in this supplement’s 12 articles which highlight key challenges; creative, evidence-based solutions; and unanswered questions. The articles address a range of pressing issues including the impact of stigma on access to care, integrating mental health into AYA HIV prevention and treatment programs, the use of mHealth interventions along the AYA HIV continuum of care, and understanding the impact of the COVID-19 pandemic on AYA HIV research and services. The papers, each co-authored by numerous AHISA teams, highlight the critical role that implementation science plays in addressing the unique needs of AYA HIV prevention, care, and treatment, and the value of networks like AHISA that provide opportunities for data sharing, learning from efforts across SSA, and exchanging ideas and strategies.

While we have efficacious tools to decrease incidence of new HIV infections and increase the percentage of HIV-positive AYA in care, we must apply these more effectively to reach this vulnerable and underserved population. It is imperative to identify and address the remaining barriers and promote scalable, empowering, and sustainable interventions that aggressively identify and enter AYAs into adolescent-friendly long-term care and treatment delivery systems. AHISA teams have consistently provided new insights about these intractable barriers, and the Alliance as a whole has supported the application of implementation science to create solutions for these often-neglected challenges. The articles in this special issue represent the collective learning of the Alliance; illustrate the value of implementation science in the context of adolescent HIV; and identify critical research gaps that should be addressed by implementation science in the future. We hope they catalyze additional interest and engagement in implementation science to advance AYA HIV prevention and care in SSA and around the globe.
